# Association study of the *CSN2* gene with milk yield in the Sapera goat

**DOI:** 10.5455/javar.2025.l981

**Published:** 2025-12-25

**Authors:** Santiananda Arta Asmarasari, Galih Ari Wirawan Siregar, Ferdy Saputra, Lisa Praharani, Zultinur Muttaqin, Diana Andrianita Kusumaningrum, Nurul Azizah, Wisri Puastuti, Dwi Yulistiani, Supardi Rusdiana, Anneke Anggraeni, Suyatno Suyatno, Teguh Ari Prabowo, Nurul Pratiwi, Cecep Hidayat

**Affiliations:** 1 Research Center for Animal Husbandry, Research Organization for Agriculture and Food, National Research and Innovation Agency (BRIN), Cibinong Science Center, Bogor, Indonesia; 2 Department of Animal Science, Faculty of Agriculture, Universitas Sumatera Utara, Medan, Indonesia

**Keywords:** Sapera goat, *CSN2* gene, association study, additive effect, dominance effects

## Abstract

**Objective:**

This research was conducted to assess the influence of *CSN2* exon 7 milk production and the additive and dominance effects.

**Materials and Methods:**

DNA was isolated from 64 goats that possessed the *CSN2* gene and subjected to a polymerase chain reaction and genotyping by Sanger sequencing.Genotype effect can be assessed through analysis of variance and the generalized linear model, which can estimate additive and dominance effects.

**Results:**

Dairy traits are greatly influenced by the crucial role played by the *CSN2* gene. The influence of SNP g.8946C > T on milk yield (MY) is statistically significant (*p* < 0.05). Nevertheless, the impact of SNP g.8956G > A on MY is not statistically significant. Furthermore, the MY of Sapera goats is affected by both Parity (*p* <0.01) and days in milk (*p* < 0.01).

**Conclusion:**

The *CC* genotype demonstrates a higher MY without noticeable additive or dominance effects. Evaluating the SNP g.8946C > T necessitates many samples and phenotypes.

## Introduction

Goat milk has gained popularity as a medicinal food due to its composition and nutritional value, including protein, minerals, vitamins, amino acids, and fatty acids, which can help boost the body’s resistance to respiratory diseases like COVID-19 compared to cow’s milk [1]. Furthermore, goat milk is considered safe for consumption by lactose intolerants due to its lower lactose content and unique digestibility [2].

In Indonesia, goat’s milk has a niche market and is less popular than cow’s milk. However, the demand for goat’s milk in Indonesia, particularly during and after the COVID-19 pandemic, has been increasing; however, its supply remains significantly lower [3]. This makes goat milk prices significantly higher than those of cow’s milk, even five to seven times higher [4].

Generally, goat milk originates from Etawah-grade (PE) goats, a local breed, crossbreeding between Etawah goats and Indonesian indigenous goats (Kacang goats). The milk production of this local breed is low, with a daily yield of 0.8–1.5 l [5]. Indonesia has introduced an exotic dairy goat breed, such as the Saanen. Based on microsatellite information, Saanen and PE goats exhibit a significant genetic difference [6,7]. Therefore, crossbreeding between Saanen and PE could potentially improve the milk production of local goats [8], resulting in a new dairy goat breed called Sapera, with an average milk production of 1.47 l/d [3].

Milk contains two primary proteins: Casein and Whey. Casein is a type of protein found in milk, containing up to 80% of the protein in goat milk, and plays several roles in nutrition and body health. Casein contains nine essential amino acids, including histidine, valine, threonine, tryptophan, lysine, leucine, isoleucine, methionine, and phenylalanine, and provides calcium for maintaining bone health. Compared to bovine milk, goat milk has higher quality and variations in composition, including lower αs1-casein and higher β-casein levels, smaller fat globules, medium-chain fatty acids, and higher mineral levels [9-11].

The encoding of casein involves four genes: *alpha-S1* (*CSN1S1*), *alpha-S2* (*CSN1S2*), beta (*CSN2*), and kappa (*CSN3*) [12]. Beta-casein, encoded by the *CSN2* gene, makes up approximately half of the total protein found in Sarda goat milk. This protein plays a vital role in the manufacturing properties of milk, particularly in the formation and stabilization of micelles in Dutch dairy goats [13]. The Goat *CSN2* promoter region has been associated with the absence of beta-casein in milk [14].

The *CSN2* locus has been known to influence the percentage of milk protein, milk fat, milk production, and the content of free fatty acids [15]. Previous research conducted by Deviandini et al*.* [16] has found SNPs in *CSN2* that influence lactose and salt content in Sapera goats. However, the effect of *CSN2* on milk yield (MY) in Sapera goats has never been studied, which is necessary to support the molecular information of an Indonesian new dairy goat breed and to complete the understanding of milk quality related to the *CSN2* gene. Therefore, this study was conducted to investigate the influence of *CSN2* exon 7 on MY and its additive and dominance effects in Sapera goats. This study will provide a genetic basis for marker-assisted selection of Sapera goats, enhancing productivity, sustainability, and economic returns in tropical dairy goat farming.

## Materials and Methods

### Ethical approval

This research was approved for ethical clearance by the Animal Welfare Animal Ethics Committee of the Agricultural Research and Development Center, registered under Balitbangtan/Balitnak/Rm/11/21.

### Animals

A total of 64 Sapera goats had blood taken from the jugular vein. The blood was stored in 10 ml EDTA Vacutainer tubes. The extraction of DNA from whole blood samples was performed using the Geneaid DNA extraction kit (Geneaid Biotech., Taiwan). The phenotypes collected for analysis were parity, days in milk (DIM), and MY. MY was recorded as daily yield (kg/day), averaged from two consecutive collections in the morning (5:00–6:00 a.m.) and afternoon (3.00–4.00 p.m.).

### DNA extraction and polymerase chain reaction (PCR)

The primer for exon 7 of the *CSN2* gene was designed based on sequences available in GenBank under the accession number AJ011018. The amplified product length from a forward primer (5'- GGC ACA GTC TCT AGT CTA TC -'3) and reverse primer (5'- CCT TTC TGC TGT ACC AGG AG -'3) was confirmed to be 418 bp. DNA was extracted using a commercially based kit to maintain high-quality genomic DNA that can be amplified. DNA concentration was determined using a spectrophotometer for both confirmation of concentration and purity before amplification by PCR.

DNA amplification was performed using the PCR method in an Applied Biosystem 9700 (Thermo Fisher Scientific, US). All PCR runs were carried out in a final volume of 25 μl, to which 2 μl of DNA, 6.1 μl of nuclease-free water, 0.3 μl of reverse primer, 0.3 μl of forward primer, and 16 μl of μl MyTaq HS Redmix (Bioline, UK) were added. Thermal cycling conditions for PCR were pre-denatured at 95°C for 5 min, with 35 cycles of denaturation for 15 sec, annealing at 60°C for 15 sec, and extension at 72°C for 10 sec, followed by final extension at 72°C for 1 min. These conditions were then optimized from preliminary experiments to achieve maximum yield and specificity of the target amplicon, as reported in the literature.

**Figure 1.**
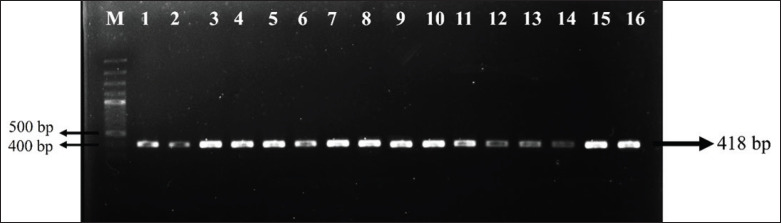
The visualization of the *CSN2* gene exon 7 in the Sapera goat.

Post-amplification, the quality of the PCR products was assessed using agarose gel electrophoresis, followed by visualization under ultraviolet light to confirm the presence of the expected 418 bp band (Fig. 1). The amplicon was sequenced using the services provided by 1st Base, located in Selangor, Malaysia, to ensure accurate identification of nucleotide variations.

### Statistical analysis

Power analysis was conducted to determine the required sample size to detect a statistically significant effect. The analysis was performed using the power package in R 4.3.1 [17]. Data analysis was conducted using R 4.3.1 [17], where the analysis of variance function was employed to compare the mean values, followed by the least significant difference post hoc range test. The statistical model for the association of *CSN2* genotype was


$$Y_{\mathrm{ijk}}=\mu +p_{i}+d_{k}+g_{j}+{ɛ}_{\mathrm{ijk}}$$

where *Y*_*ijk*_ was MY, μ was the population mean, *p*_*i*_ was the parity effect, *d*_*k*_ was the DIM effect, *g*_*i*_ was the genotype effect, and ε_*ijk*_ was an error.

The generalized linear model was used to analyze the additive and dominance effects:


$$y=\mu +C_{\mathrm{pp}}+C_{\mathrm{ii}}+C_{\mathrm{aa}}+C_{\mathrm{dd}}+e$$

where *y* is the MY,μ is the intercept, *C_pp_* is the covariate coefficient of parity, *C_ii_* is the covariate coefficient of DIM, *C_aa_* is the covariate coefficient of additive effect, the *C_dd_* is the covariate coefficient of dominance effect, and *e* is the residual standard error.

## Results

The findings indicated a significant effect (*p* < 0.05) of SNP g.8946C > T on milk production. (Table 1). The milk production of the *CC* genotype at SNP g.8946C > T outperforms the *CT* and *TT* genotypes. The MY is not significantly influenced by the SNP g.8956G > A. The DIM in this study were 223 ± 58 days with a parity of 1.95 ± 0.74. Meanwhile, the average milk production per population was 0.83 ± 0.37 kg/day.

**Table 1.** Effect g.8946C > T and g.8956G > A on milk traits in Sapera goat.

	g.8946C > T			g.8956G > A	
Genotype (*n*)	CC (13)	CT (28)	TT (23)	GG (41)	GA (23)
MY (kg/day)	1.028 ± 0.456^a^	0.753 ± 0.339^b^	0.81 ± 0.3^ab^	0.833 ± 0.388^a^	0.831 ± 0.333^a^

Different superscripts in the same row showed significant differences (*p* < 0.05).

The parity, DIM, additive effects, and dominance effects on the average MY are shown in Table 2. The data show that the MY is significantly (*p* < 0.01) influenced by parities and DIM. Data on the additive and dominance effect showed no significant differences between MY with the *C* allele and those with the *T* allele.

**Table 2.** Additive and dominance effect of g.8946C > T, on MY (kg/day).

Parity	DIM	Additive	Dominance
0.185 ± 0.063**	0.002 ± 0.001**	0.044 ± 0.078	−0.045 ± 0.165

**p < 0.01

Table 3 shows the power analysis for SNP Effects on Milk Production Traits. Table 4 shows Haplotype Associations with MY in Sapera Goats. The power analysis revealed distinct differences between SNP1 and SNP2 in their associations with productive and reproductive traits. For SNP1, moderate-to-large effect sizes were observed in MY and parity, particularly in the comparisons between *CC* versus *CT* and *CC* versus *TT* genotypes. However, the current sample sizes were insufficient to achieve adequate statistical power, with most comparisons showing power below 0.6. For instance, MY showed an effect size of *d* = 0.725 for *CC* versus *CT*, yet only reached a power of 0.56, indicating that at least 51 animals per genotype group would be required to achieve 80% power. Similarly, parity exhibited moderate effect sizes (0.53–0.55), but observed power remained below 0.42. In contrast, DIM showed only small effect sizes across all genotype comparisons, with power below 0.20.

**Table 3.** Power analysis for SNP effects on milk production traits.

Trait	SNP	Comparison	Cohens_d	N1	N2	Power	Total *N* required
MY	g.8946 C > T	*CC* vs *CT*	0.725	13	28	0.558	62
MY	g.8946 C > T	*CC* vs *TT*	0.561	13	23	0.349	102
MY	g.8946 C > T	*CT* vs *TT*	−0.198	28	23	0.106	800
MY	g.8956 G > A	*GA* vs *GG*	−0.006	23	41	0.050	828454

**Table 4.** Haplotype associations with MY in Sapera goats.

Trait	Haplotype	*N*	Mean ± SE	*p* -value
MY	*CC GG*	7	1.155 ± 0.212	0.212
MY	*CC_GA*	6	0.879 ± 0.109	0.212
MY	*TT_GA*	8	0.873 ± 0.159	0.212
MY	*TT_GG*	15	0.789 ± 0.060	0.212
MY	*CT_GA*	9	0.761 ± 0.091	0.212
MY	*CT_GG*	19	0.750 ± 0.085	0.212

## Discussion

This study aimed to investigate the impact of the *CSN2* exon 7 on milk production, focusing on the identified SNPs g.8946C > T and g.8956G > A. The g.8946C > T SNP was found to be a non-synonymous mutation that alters the amino acid sequence from alanine (GCA) to valine (GTA) at position 177. In contrast, the g.8956G > A SNP is a synonymous mutation, preserving the amino acid glutamine at position 180. This distinction among the mutations is significant, given that non-synonymous mutations are more likely to affect phenotypic traits due to altering protein structure and function. Functional differentiation is a concept of great biological significance. Non-synonymous mutations are more likely to affect protein conformation, stability, or intermolecular interactions, thereby influencing phenotypic traits.

Conversely, synonymous mutations tend to have reduced or context-dependent effects, often mediated through changes in codon usage or mRNA stability [18]. The presence of four established mutations in the *CSN2* exon 7, at amino acid residues 58, 166, 167, and 177 [19], as identified in previous research, further establishes the genetic heterogeneity in this region. The β-casein, which is encoded by CSN2, plays a significant role in milk’s calcium transport and micelle-forming processes [14]. Structural modifications, such as the substitution of alanine with valine (Ala�Val), may influence hydrophobic interactions and the conformation of local structures, potentially leading to alterations in micelle dimensions and the efficiency of secretion. Such alterations can disrupt the equilibrium between the volume of milk produced and the partitioning of solids. Indeed, previous research has demonstrated that genetic variation within the *CSN2* gene is correlated with the compositional and technological attributes of milk across various goat breeds [12,13].

Consequently, the functional polymorphism g.8946C > T aligns with established biological mechanisms that connect casein variants to lactation performance. This study suggests practical value in using SNP g.8946C > T as a potential molecular marker. Interestingly, our findings align with earlier research by Deviandini et al*.* [16], who also identified the significance of the g. 8946C > T SNP in milk production traits. The prevalence of the *CC* genotype, particularly in Banat’s White Dairy Goat Breeds, is 0.73 and is associated with superior dairy performance [20]. The broader literature further supports this result: in Awassi sheep, *CSN2* genotypes were strongly linked to MY and composition [21,22], while in Sarda goats, casein loci have repeatedly been implicated in fat and protein yields [12]. Taken together, these data emphasize the importance of exon 7 as a hotspot for functional polymorphism within the casein cluster.

The investigation revealed that while the genotype had an observable influence on milk production, the additive and dominance effects associated with these SNPs were not statistically significant. This lack of highly additive or dominant effects suggests that other loci or environmental influences are more significant in regulating milk production. Additionally, the stronger effect of haplotypes at *CSN1S1* and *CSN3* compared to those at *CSN1S2* and *CSN2* underscores the complex interaction of several genetic factors in regulating milk traits. This level of complexity is also evident in the findings for the impact of the *CSN2* gene on milk protein and fat in Sarda and Awassi goat breeds, again supporting the role of several loci in overall milk production traits [12,21].

In addition, the significant effect of parity and DIM on milk production discovered in this study aligns with published literature. Parity increase is generally found to be associated with increased MY, as multiparous animals tend to exhibit improved lactation performance compared to primiparous animals [23]. They have improved physiological adaptation and lactation experience. Our results indicate that lactation stage, particularly in goats with four or more parities, increases total MY, further supporting this argument, which concurs with the finding of Gafsi et al*.* [24].

Furthermore, the outcomes in the first 100 days of lactation, with higher MYs, underscore the importance of first lactation performance, which is essential for achieving the highest overall milk production [25]. Genetic merit is key during the initial phase, the essence behind the need for genetic improvement initiatives, and the traits that support the first lactation [26]. Management practices, such as kidding season and feeding, have significant effects on dairy goat performance, interacting with parity and DIM. Optimistic nutritional management improves MY, persistency, and metabolic health, and multiparous goats also utilize feed more efficiently compared to primiparous goats [5].

In conclusion, this study brings important contributions to the genetic origin of milk production traits in goats. The contribution of the *CSN2* exon 7 gene and its SNPs to milk yield, in addition to the pivotal influence of parity and DIM, highlights the complex nature of dairy production. Future studies should continue to investigate these relationships, perhaps with increased sample sizes and phenotypic diversity to gain further insight into the intricate genetic architecture of milk production traits.

Two mutations were identified in this study, specifically at amino acid positions 177 and 180. In Czech Dairy Goat Breeds, it is known that the *CC* genotype in mutation 8946 has the highest frequency of 0.496 [27]. In Indian sheep, *CSN2* is known to have 15 *CSN2* protein variations and 21 non-synonymous SNPs [28]. The diversity of the *CSN2* gene in sheep is greater than in goats.

Although the genotype had an influence, the additive and dominance effects were not significant. Furthermore, Haplotypes at the *CSN1S1* and *CSN3* loci have a greater effect on dairy traits than those at the *CSN1S2* and *CSN2* loci [29]. The milk protein and fat composition in Sarada goats is influenced by the *CSN2* gene [12]. In Awassi goats, the *CA* genotype is linked to significant milk production of 165.2 ± 22.0 kg, with *CSN2* playing a crucial role [21]. *CSN1S1* and *CSN3* in Norwegian goats have an additive effect on fat percentage, protein percentage, and MY. Notably, the dominance effect is particularly significant in protein and fat percentage [30]. Another study concluded that the *CSN3* variants have a slight impact on daily milk production [31].

The study revealed that both parity and DIM had a significant impact on MY. The milk quantity is influenced by both parity and breed, as those that have given birth multiple times tend to yield a greater MY [22,32]. The extended lactation length of goats, especially those at parity ≥4, leads to a notable increase in milk production [33]. Moreover, DIM had an impact on the MY. The initial 60 days of lactation demonstrated a higher MY, which can be attributed to the genetic merit and the sustainable milk production in Syrian Damascus goats [34].

## Conclusion

The *CC* genotype yields more milk than other genotypes. However, no additive or dominant effects are observed. A large quantity of samples and phenotypes is required for the assessment of the g.8946C > T SNP. Additionally, parity and DIM have a strong impact on MY, indicating that animals with higher parity or longer lactation periods tend to produce more milk. This study highlights the importance of understanding both genetic variation and non-genetic factors, such as parity and DIM, in improving milk production performance. The variation in the *CSN2* gene, particularly SNP g.8946C > T, can serve as a reference for genetic selection to enhance milk production in the future. The identification of g.8946C > T as a potentially informative marker provides a valuable reference for marker-assisted and genomic-assisted selection in tropical dairy goat systems.

## List of abbreviations

ANOVA, analysis of variance; CSN, Casein; DIM, days in milk; DNA, deoxyribonucleic acid; EDTA, Ethylenediamine-tetraacetic acid; PCR, polymerase chain reaction; PE, Etawah grade; SNP, single-nucleotide polymorphism.
